# Antibiotics induced commensal flora disruption favours *escherichia coli *AIEC colonization and mesenteric translocation in NOD2 knock-out mice

**DOI:** 10.1186/1479-5876-9-S2-O8

**Published:** 2011-11-23

**Authors:** Maryline Drouet, Cécile Vignal, Elisabeth Singer, Luc Dubreuil, Pierre Desreumaux, Christel Neut

**Affiliations:** 1 INSERM U995, Lille, France; 2 Faculty of Pharmacy, Lille 2 University, Lille, France; 3 Dept. of Gastroenterology, Claude Huriez Hospital, Lille, France

## Background

Ileal lesions of Crohn's disease (CD) patients are colonized by pathogenic adherent-invasive *Escherichia coli* (AIEC). NOD2 gene mutations are risk factors for ileal CD and are associated with an abnormal AIEC-induced immune response.

## Aim

Since innate immune system and commensal microbiota are critical to maintain intestinal barrier integrity, we evaluated the impact of commensal microbiota disruption induced by short term antibiotic treatment on AIEC colonization and translocation in wild type (WT) and NOD2 knock-out mice (NOD2KO).

## Methods

Disruption of commensal microbiota was induced by a 3 days antibiotic treatment orally administered in WT and NOD2KO mice. At day 3, mice were infected with 10^9^ CFU AIEC once a day for 2 days. Animals were sacrificed at day 1, 5, 30 and 60 after AIEC administration. Ileum, colon and mesenteric lymph nodes (MLN) were sampled for AIEC quantification in ileal and colonic tissues, bacterial translocation in MLN, and evaluation of histological abnormalities and intestinal inflammation.

## Results

Without antibiotic treatment, AIEC was not able to colonize WT and NOD2KO mice. Compared to control animals, commensal microbiota disruption induced by antibiotics led to a significant increase of ileal and colonic adherent AIEC in both WT and NOD2KO mice at day 1. Persistent AIEC colonization was still observed until day 30 in the ileum but not in the colon of NOD2KO mice, disappearing at day 60. Mesenteric translocation of AIEC was observed until day 30 only in NOD2KO mice. No histologic ileal and colonic abnormalities and/or inflammation were observed in WT and NOD2KO mice treated with antibiotics and infected with AIEC.

## Conclusion

Sustained AIEC colonization in ileal tissues of mice was induced by commensal flora disruption promoted by short term antibiotic treatment, independently of NOD2 expression. In contrast, commensal flora disruption by antibiotics induced a long term AIEC bacterial translocation in a NOD2 dependent manner.

